# Increased Requirement of Replacement Doses of Levothyroxine Caused by Liver Cirrhosis

**DOI:** 10.3389/fendo.2018.00150

**Published:** 2018-04-18

**Authors:** Salvatore Benvenga, Giovanni Capodicasa, Sarah Perelli, Silvia Martina Ferrari, Poupak Fallahi, Alessandro Antonelli

**Affiliations:** ^1^Department of Clinical and Experimental Medicine, University of Messina, Policlinico Universitario G. Martino, Messina, Italy; ^2^Master Program on Childhood, Adolescent and Women’s Endocrine Health, University of Messina, Policlinico Universitario G. Martino, Messina, Italy; ^3^Interdepartmental Program of Molecular & Clinical Endocrinology, and Women’s Endocrine Health, University Hospital, Policlinico Universitario G. Martino, Messina, Italy; ^4^Department of Clinical and Experimental Medicine, University of Pisa, Pisa, Italy

**Keywords:** liver cirrhosis, undertreated hypothyroidism, thyroxine malabsorption, liquid levothyroxine, thyroxine-binding globulin

## Abstract

**Background:**

Since hypothyroidism is a fairly common dysfunction, levothyroxine (L-T4) is one of the most prescribed medications. Approximately 70% of the administered L-T4 dose is absorbed. The absorption process takes place in the small intestine. Some disorders of the digestive system and some medicines, supplements, and drinks cause L-T4 malabsorption, resulting in failure of serum TSH to be normal. Only rarely liver cirrhosis is mentioned as causing L-T4 malabsorption.

**Case report:**

In this study, we report increased requirement of daily doses of l-thyroxine in two patients with the atrophic variant of Hashimoto’s thyroiditis and liver cirrhosis. In one patient, this increased requirement could have been contributed by the increased serum levels of the estrogen-dependent thyroxine-binding globulin (TBG), which is the major plasma carrier of thyroid hormones. In the other patient, we switched from tablet L-T4 to liquid L-T4 at the same daily dose. Normalization of TSH levels was achieved, but TSH increased again when she returned to tablet L-T4.

**Conclusion:**

Liver cirrhosis can cause increased L-T4 requirements. In addition to impaired bile secretion, the mechanism could be increased serum TBG. A similar increased requirement of L-T4 is observed in other situations characterized by elevation of serum TBG. Because of better intestinal absorption, L-T4 oral liquid formulation is able to circumvent the increased need of L-T4 in these patients.

## Introduction

Hypothyroidism is a fairly common dysfunction and its incidence grows with age. Therefore levothyroxine (L-T4) is one of the most prescribed medications (1). Approximately 70% of the administered L-T4 dose is absorbed after L-T4 tablet dissolution in the acid intragastric environment. The absorption process takes place in the duodenum, jejunum, and ileum (2–4).

Several gastrointestinal disorders (gastritis, celiac disease, lactose intolerance, intestinal parasitosis, and bariatric surgery) and various medicines, supplements, and drinks reduce the absorption of L-T4 (5–14). Recently, Lobasso et al. (15) reported a woman with systemic sclerosis in whom dysmotility of distal esophagus caused L-T4 malabsorption. Also, a dysbiosis has been postulated to be involved in pharmacologic homeostasis of thyroxine (16, 17).

Among 210 subjects who were observed by one of us because of undertreated primary hypothyroidism in spite of adequate daily dose of L-T4, 27 (12.9%) had an increased need of L-T4 (5). The gastrointestinal-related causes of increased need of L-T4 were celiac disease (*n* = 3), Crohn’s enteritis (*n* = 1), co-ingestion of proton-pump inhibitors (PPIs, *n* = 6) alone or combined with other L-T4 sequestrants (ferrous salts or calcium salts). There are two disorders of the digestive system, liver cirrhosis, and diabetic diarrhea, which are rarely mentioned as the cause of an increase of L-T4 demand (18, 19). Chronic obstructive liver disease and pancreatic insufficiency are mentioned only in less recent reviews (20, 21). Textbooks and literature fail to mention that bile is important to maximize the intestinal absorption of L-T4 (22).

## Background

Target serum TSH levels have to be used to monitor L-T4 therapy in primary hypothyroidism (1). The target TSH in primary hypothyroidism “*should be the normal range of a third generation TSH assay*” or, if unavailable, should be 0.45–4.2 mU/l. However, according to the reference population of the National Health and Nutrition Examination Survey III, the normal values are 0.45–5.90 mU/l in the 70- to 79-year-old and 0.33–7.50 mU/l in ≥80-year-old patients. By contrast, the 2003 National Academy of Clinical Biochemistry Laboratory and Medicine Practice Guidelines suggest that the upper limit of normal range is 2.5 mU/l (23).

A complete diagnostic work-up for failure of serum TSH to become normal is multidisciplinary, may require hospitalization and be expensive. The work-up includes several steps, whose number and order may vary in the individual patient (24). The initial steps involve confirmation of diagnosis and laboratory data, assessment of patient compliance, and control of the formulations taken. Subsequent steps involve investigation of incorrect ingestion of L-T4, causes of malabsorption, including increased L-T4 turnover or excretion; if necessary, l-thyroxine absorption test is performed. A similar approach has been proposed by one of us elsewhere (5).

Here, we report two cases of undertreated hypothyroidism, causing increased requirement of daily doses of l-thyroxine, in patients with cirrhosis of the liver. In one patient, tablet L-T4 was replaced by oral liquid L-T4, which normalized serum TSH.

### Report of the First Case

A 54-year-old man with hepatitis C virus-related liver cirrhosis had been diagnosed autoimmune hypothyroidism (atrophic variant of Hashimoto’s thyroiditis) approximately 8 years earlier, which was treated with 100 μg/day L-T4. Serum TSH had been consistently ≤3.4 mU/l, until symptomatology characterized by asthenia, weight loss, and yellowish staining of the sclera appeared. This led to the diagnosis of liver cirrhosis. Serum TSH increased to 9.3 mU/l so that L-T4 was progressively increased to 150 μg/day. However, serum TSH did not fall below 6.6 mU/l. When observed, the patient had scleral jaundice, bilateral gynecomastia and was treated with PPI (lansoprazole), a class of drugs that reduces the absorption of L-T4 by increasing gastric pH (3–8, 25–27). However, lansoprazole was taken discontinuously. Because of gynecomastia, serum testosterone, estradiol (E2), and prolactin (PRL) had been requested before our observation.

#### Results

Total testosterone was borderline low (300 ng/dl, reference range 290–960), and E2 mildly elevated (53 pg/ml, reference range for men <40), as it was PRL (24 ng/ml, reference range for men <16). Knowing that thyroxine-binding globulin (TBG) is the estrogen-upregulated and androgen-downregulated major thyroid hormone plasma carrier (28–34) and that circulating TBG increases in liver diseases (28, 35–41), we complemented the PRL and sex hormone assays with TBG assay. We hypothesized that, similarly to the increased requirements of L-T4 replacement therapy during gestation (a physiological hyperestrogenic state in which TBG is increased) (42, 43) but unlike the decreased requirements of L-T4 replacement therapy associated with hyperandrogenism (when serum TBG decreases) (34), elevation of serum TBG (and associated increased binding of L-T4 to it) might have contributed to undertreated hypothyroidism in our patient. Indeed, serum TBG was high (60 µg/ml; reference range 12–32), a level comparable to that of pregnant women. The patient was soon lost at follow-up. However, 5 years later, one of his relatives called over the telephone to have a second opinion concerning the combined L-T4 + L-T3 therapy. On that occasion, we learned that in our patient cirrhosis had been managed successfully by liver transplantation and that gynecomastia disappeared. Although he continued to take discontinuously PPI (6 h after L-T4), his latest serum TSH was 2.4 mU/l (reference range 0.27–4.2) under 100 μg/day L-T4.

### Report of the Second Case

The second cirrhotic patient is a 64-year-old hypothyroid woman with the atrophic variant of Hashimoto’s thyroiditis under treatment with tablet LT-4 over the last 10 years. She was euthyroid upon taking 150 μg/day L-T4, which was taken 30 min before breakfast. Twelve months after the last endocrinological control, she was diagnosed with hepatitis C virus-related cirrhosis. After 7 months, laboratory tests showed subclinical hypothyroidism (TSH = 8.65 mU/l, FT4 = 9.5 pg/ml; Table 1), although her body weight had not undergone significant changes.

**Table 1:** Thyroid parameters measured when patient no. 2 was receiving oral levothyroxine (L-T4) in the tablet formulation, after 2 months of L-T4 in the liquid formulation (in bold), and then after 1 month of return to the tablet formulation.^a^

L-T4 formulation	TSH, mU/l	FT3, pg/ml	FT4, pg/ml
Tablet	8.65	2.7	9.5
**Liquid**	**3.03**	**3.0**	**12.3**
Tablet	6.37	2. 9	11. 8

*^a^Because body weight of this patient was stable (67–68 kg) over time, the daily dose of 150 μg/day was also stable when normalized for body weight, namely, 2.23 μg/kg b.w./day*.

*Reference ranges are 0.3–3.5 mU/l (TSH), 2.3–4.2 pg/ml (FT3), and 8–17 pg/ml (FT4). The tabulated hormone levels were measured at observation when the patient was under tablet L-T4, after 2 months from switch to oral liquid L-T4 and 1 month after switch back to tablet L-T4*.

#### Results

Since we are aware of a better absorption profile of novel formulations of L-T4 over the classic tablet formulation (see Discussion), we switched tablet L-T4 to oral liquid L-T4, without changing the daily dose. TSH levels became normal after about 60 days (3.0 mU/l), and both FT4 and FT3 levels increased from close to the lower normal limit to close to the mid-range value (12.3 and 3.0 pg/ml) (Table 1). Accordingly, she felt better. To confirm that this improvement was not random, L-T4 was switched back to the tablet formulation at the same dosage. One month after this switch, serum TSH bounced back to abnormal levels, in parallel with a decline of both FT4 and FT3 levels (Table 1).

## Discussion

Most patients with hepatitis C virus develop at least one extrahepatic manifestation, including autoimmune thyroid disease (44). Accordingly, hypothyroidism and thyroid autoimmunity are significantly more frequent in HCV patients with respect to controls (44).

The two patients reported here remind us that liver cirrhosis is one of the causes of undertreated hypothyroidism and associated greater L-T4 requirement. The increased L-T4 requirement associated with liver cirrhosis could be due to defects in bile production and excretion. Indeed, bile is important to maximize the intestinal absorption of L-T4 (22). However, one additional mechanism for the increased dosage of L-T4 could be the same as that occurring in pregnancy or other conditions of hyperestrogenism (28, 29, 32, 33), namely, increased binding of L-T4 by the increased estrogen-driven serum levels of TBG, the major thyroid hormone plasma carrier, and failure to compensate for this increased binding. Elevation of serum E2 in cirrhosis and E2 normalization after liver transplantation was shown by other authors (41, 45). Congruent with our data on the second patient, an Italian study found that the increased L-T4 requirement occurring during gestation is significantly less frequent if hypothyroid pregnant women are supplemented with liquid L-T4 compared with tablet L-T4 (1/14 vs 7/17, *P* = 0.038) (46). An increased requirement of L-T4 is more likely to occur in pregnant women with no residual thyroid function (e.g., because of radioablation, thyroidectomy), compared with women with non-atrophic Hashimoto’s thyroiditis (42, 43). Non-iatrogenic conditions of absent functional thyroid tissue are thyroid agenesia or, like our patients, thyroid atrophia.

In the first cirrhotic patient described here, co-ingestion of one medication (a PPI) that is known to interfere with L-T4 intestinal absorption (5–8) is unlikely to be the cause of undertreated hypothyroidism. In the Sachmechi et al. study (25), 37 euthyroid patients who had received stable L-T4 replacement for at least 6 months and in whom lansoprazole therapy (the same PPI taken by our patient) was later initiated, serum TSH levels were measured before and at least 2 months after the PPI was started. Of the 37 patients, only 7 (19%) had post-PPI TSH levels >5 mU/l, requiring a mean increase of 20 μg/day L-T4 (+35%). By contrast, short-term (47, 48) and, presumably, discontinuous treatment with PPI (as in our patient) has no impact on L-T4 intestinal absorption.

The second patient permits to add the chronic liver disease setting to the other settings (5, 6, 18, 26, 27, 49–63) in which novel formulations of L-T4, because of their more favorable pharmacokinetics profile, perform better than the classic tablet formulation in achieving target levels of TSH (64). From a gastroenterological perspective, settings of interest are the correction of the impaired tablet L-T4 absorption caused by food and beverages, anti-ulcerants (26, 27), esophageal dysmotility (15), gastritis (58, 59), enteral feeding (63), and bariatric surgery (54, 55).

In sum, liver cirrhosis can cause undertreated primary hypothyroidism and associated increased L-T4 requirement. The L-T4 increased requirement is due to impaired bile secretion and increased serum TBG resulting in increased TBG-T4 binding. This augmented TBG-T4 binding cannot be compensated by increased free T4 levels originating from an increased thyroid gland secretion, if the patient has hyperestrogenism (which would raise serum TBG levels even more) and has no or minimal functioning thyroid tissue. A similar increased requirement of L-T4 is typically observed during gestation (a physiologic condition of elevated serum estrogens and serum TBG), especially in pregnant women with non functioning thyroid tissue, when serum TBG is elevated.

This study suggests that, further to the known situation in hypothyroid pregnant women, elevated serum levels of the major plasma carrier of thyroid hormones (TBG) increase the daily requirement of L-T4 also in hypothyroid men with an atrophied thyroid. Like in iatrogenic conditions of an entirely non-functional thyroid gland (i.e., radioablation and thyroidectomy), the increased binding of L-T4 to circulating TBG cannot be compensated by an increased hormone output by the thyroid.

## Concluding Remarks

Our report highlights live cirrhosis as one cause of increased requirement for L-T4. Hepatic dysfunction may involve the rise in TBG concentrations, like in pregnancy. Our study is also the first showing that the L-T4 oral liquid formulation can solve the problem of the increased need of tablet L-T4 dose in liver cirrhosis because of better intestinal absorption.

## Ethics Statement

The patients gave written informed consent for the publication of this case report.

## Author Contributions

All the authors contributed equally to recruited patients and wrote the present work.

## Conflict of Interest Statement

IBSA Farmaceutici Italia s.r.l. and IBSA Institut Biochimique SA (Lugano, Switzerland) provided SB and AA with novel formulations to perform clinical studies. However, IBSA had no role in any phase of writing this paper.
